# Analysis of Binary Multivariate Longitudinal Data via 2-Dimensional Orbits: An Application to the Agincourt Health and Socio-Demographic Surveillance System in South Africa

**DOI:** 10.1371/journal.pone.0123812

**Published:** 2015-04-28

**Authors:** Maria Vivien Visaya, David Sherwell, Benn Sartorius, Fabien Cromieres

**Affiliations:** 1 Department of Pure and Applied Mathematics, University of Johannesburg, Johannesburg, South Africa; 2 School of Computational and Applied Mathematics, University of the Witwatersrand, Johannesburg, South Africa; 3 Discipline of Public Health Medicine, School of Nursing and Public Health, University of KwaZulu-Natal, Durban, South Africa; 4 Graduate School of Informatics, Kyoto University, Kyoto, Japan; Örebro University, SWEDEN

## Abstract

We analyse demographic longitudinal survey data of South African (SA) and Mozambican (MOZ) rural households from the Agincourt Health and Socio-Demographic Surveillance System in South Africa. In particular, we determine whether absolute poverty status (APS) is associated with selected household variables pertaining to socio-economic determination, namely household head age, household size, cumulative death, adults to minor ratio, and influx. For comparative purposes, households are classified according to household head nationality (SA or MOZ) and APS (rich or poor). The longitudinal data of each of the four subpopulations (SA rich, SA poor, MOZ rich, and MOZ poor) is a five-dimensional space defined by binary variables (questions), subjects, and time. We use the orbit method to represent binary multivariate longitudinal data (BMLD) of each household as a two-dimensional orbit and to visualise dynamics and behaviour of the population. At each time step, a point (*x*, *y*) from the orbit of a household corresponds to the observation of the household, where *x* is a binary sequence of responses and *y* is an ordering of variables. The ordering of variables is dynamically rearranged such that clusters and holes associated to least and frequently changing variables in the state space respectively, are exposed. Analysis of orbits reveals information of change at both individual- and population-level, change patterns in the data, capacity of states in the state space, and density of state transitions in the orbits. Analysis of household orbits of the four subpopulations show association between (i) households headed by older adults and rich households, (ii) large household size and poor households, and (iii) households with more minors than adults and poor households. Our results are compared to other methods of BMLD analysis.

## Introduction

Binary multivariate longitudinal data (BMLD) is here exemplified by the binary responses in a Yes/No form to a set of *p* ≥ 1 questions (variables) asked to each subject of a (sample) population over a period of time. As in the convenient convention of binary coding of 0 for a negative response and 1 a for positive response [1], the outcome of each of the binary variables here is coded as 0 if the outcome is unfavourable (by hypothesis) to a given purpose, and 1 if favourable.

Many BMLD studies use regression techniques [2] or Markov, transition and forecasting models ([3–5]). These methods involve parameter estimation for the explanatory variables. However, visual analysis of data is equally important as it presents initial insights about the data. Descriptive tools such as tables and charts give a visual summary and simpler interpretation. For visual analysis of multivariate longitudinal data, some analysis is given in ([6, 7]) but very few tools are available when the data is binary.

In [8], the focus is on visualizing the complex border between patterns of BMLD. The border in a multidimensional space is converted into visual 2-dimensional and 3-dimensional forms. However, it does not illustrate patterns and dynamics of the population over time. A technique that accounts for dynamics of BMLD and within subject information is the orbit method discussed in [9]. Orbit method here is distinct from the Kirillov orbit method used in representation theory [10]. By an *orbit* we mean a sequence of points related by the evolution function of the underlying system. The method of orbit is a technique based on deterministic outcomes with emphasis on geometric visualization of multivariate longitudinal data as 2-dimensional orbits. It considers the frequency of change of variables and uses the order of variables for constructing orbits that represent subjects from the population. Orbits give insight for data analysis and provide exact data visualization.

Here we use the orbit method to analyse binary demographic data of households from the Agincourt Health and Socio-Demographic Surveillance System (AHDSS) in South Africa. The longitudinal AHDSS data have been studied e.g. in [11] and [12] where a spatial-temporal model to analyse distribution of mortality and asset accumulation rate respectively were employed. Determinants of socio-economic status/poverty or the relationship between poverty and increased mortality were viewed from more of a static perspective i.e. not from the more dynamic approach offered by the orbit. The orbit approach presents a visualisation of the data in a truly longitudinal-temporal manner. The orbit method is briefly illustrated in [9] using variables regarding child educational progression in AHDSS. However, detailed interpretation of subsets in the subspace, nor analysis of orbits in the space, were not discussed. Aside from [9], we know of no other visual analysis employed for AHDSS, particularly involving household variables pertaining to socio-economic determination.

The AHDSS longitudinal data analysed here is of about 4,000 households from 2001–2007. With purpose

$$\begin{array}{l} Purpose\;:\;To\;determine\;the\;association\;of\;absolute\;poverty\;status\\ with\;selected\;household\;variables, \end{array}$$

we consider the following questions:

$$\begin{array}{c} \begin{array}{cc} Q_{0}\left(\text{HH}\right): & \text{Household}\;\text{head}\;\text{age}\geq 40\;\text{years}\;\text{old}?\\ Q_{1}\left(\text{HS}\right): & \text{Household}\;\text{size}\geq 3\;\text{individuals}?\\ Q_{2}\left(\text{HD}\right): & \text{Cumulative}\;\text{household}\;\text{deaths}\;\text{low}(\text{excluding}\;\text{household}\;\text{head}\;\text{death})?\\ Q_{3}\left(\text{AM}\right): & \text{More}\;\text{adults}(\text{age}\geq 18)\text{than}\;\text{minors}?\\ Q_{4}\left(\text{IF}\right): & \text{Internal}\;\text{influx}\;\text{negative}\\ & \left(i.e.\text{more}\;\text{people}\;\text{migrating}\;\text{into}\;\text{the}\;\text{household}\;\text{than}\;\text{leaving}\right)\\ Q_{5}\left(\text{HN}\right): & \text{Household}\;\text{head}\;\text{nationality}-\text{South}\;\text{African}\;\text{or}\;\text{former}\;\text{Mozambican}\;\text{refugee}?\\ Q_{6}\left(\text{APS}\right): & \text{Absolute}\;\text{poverty}\;\text{status}-\text{below}\;\text{poverty}\;\text{line}\;\text{or}\;\text{above}\;\text{poverty}\;\text{line}? \end{array} \end{array}$$ (2)

Each question in (Eq 2) is associated to a variable, e.g. *Q*_0_ to household head age (HH), *Q*_1_ to household size (HS), and so on. We will sometimes refer to question *Q*_*i*_ (*i* = 0, 1, …, 6) as variable *i*.

The advantage in representing the data of each subject as a two-dimensional orbit is that orbits capture the dynamics of change in response of each subject so it reveals information of change over time at both individual and population level while retaining the full information of the original data. Using orbits for data analysis give a way to visualize data, i.e. identify clusters associated to stable (less frequently changing) variables, and patterns in subpopulations associated to clusters. BMLD can involve hundreds of variables so visualising in *d* ≥ 4 dimensions is difficult. Survey data (e.g. in the social sciences) is usually large both in dimension and in size but orbit representation is feasible for large numbers of variables and subjects. In this application of orbits to the analysis of AHDSS survey data, we hope to contribute in giving new insights in the analysis of binary multivariate longitudinal data.

## Materials and Methods

### Description of Data

The Agincourt Health and Socio-Demographic Surveillance System (HDSS) is located in Bushbuckridge in northeast South Africa and was established in 1992. Bushbuckridge is a poor rural sub-district that is made up of South African and former Mozambican refugees (approximately a third of the population) [13, 14]. There have been annual updates of births, deaths, in- and out-migrations of individuals identified as members of households, as well as regular special modules (e.g. household asset ownership) used to derive a socio-economic status index.

Recall our purpose and questions given in (Eq 1) and (Eq 2). Regarding absolute poverty status (APS), it is independent of the household variables associated to questions in (Eq 2). Here, households above the absolute poverty line was defined using the definition proposed in [15] for a sub-Saharan African setting, namely ownership of a radio and bicycle, a cement floor in the house, and access to public water and a pit latrine (toilet). Absolute poverty classification is thus independent of the 6 explanatory variables used in our orbit analysis. APS of households below the poverty line are coded 0, while APS above the poverty line is coded 1. Because APS is gathered only in 4 out of the 7 observation years (i.e. at *t* = 2001, 2003, 2005, and 2007), we use the mean APS of a household over 7 years, which we denote by $\bar{APS}$. If $\bar{APS}\in$[0, 0.5], then $\bar{APS}$ is coded 0. Otherwise, $\bar{APS}$ is coded 1. Our sample population consists of 7715 household units, 4158 of which are either always above or below the absolute poverty line for all four years that APS was gathered. For these households, APS information not gathered for the three years 2002, 2004, and 2006 will not affect the coding of their $\bar{APS}$. Households with $\bar{APS}$ = 0 are referred to as Poor households, and households with $\bar{APS}$ = 1 as Rich households. Our analysis will only consider these 4158 households. Ethical clearance for the primary study was given by the University of the Witwatersrand Human Research Ethics Committee (Medical). The data used in this study does not contain clinical records (nor does the core Agincourt HDSS database). Individual and household identifiers are anonymized/de-identified by the data managers prior to handing it over to researchers for analysis to ensure confidentiality.

Aside from $\bar{APS}$, household head nationality is also *constant* throughout the survey period. In addition, former Mozambican (MOZ) refugees experience significantly higher levels of poverty compared to their South African (SA) counterparts and this gap has persisted over time [12, 16, 17]. It is then useful to extract *Q*_5_ and *Q*_6_ and analyse by these subpopulations of households where both poverty status and household head nationality are unchanging. We divide our population into four subpopulations, namely SA Rich, SA Poor, MOZ Rich, and MOZ Poor. Each subpopulation is analysed using *p* = 5 variables associated to *Q*_0_ to *Q*_4_. Binary data of the four subpopulations is given in S1 Dataset. From [12, 16], a ‘yes’ answer to questions *Q*_0_ to *Q*_4_ is assumed to be favourable to APS so we code all yes = 1, and all no = 0. Table 1 gives the favourable and unfavourable code for each of the five questions. Table 2 gives the number of households by constant variables (i.e. nationality and $\bar{APS}$).

**Table 1:** Favourable(= 1) and unfavourable(= 0) answer to questions *Q*_0_ to *Q*_4_.

*Q*_0_ (HH: household head)	*Q*_1_ (HS: household size)	*Q*_2_ (HD: household death)	*Q*_3_ (AM: adult(A) to minor(M) ratio	*Q*_4_ (IF: internal influx)
HH<40 = 0	HS<3 = 0	HD high = 0	A<M = 0	IF^+^ = 0
HH≥40 = 1	HS≥3 = 1	HD low = 1	A≥M = 1	IF^−^ = 1

**Table 2:** Distribution of households by nationality and mean APS.

Population	SA Rich	SA Poor	MOZ Rich	MOZ Poor
4158	2610	421	468	659

### The Method of Orbits

Given the number of variables *p* ≥ 1, denote by

$$\begin{array}{c} M_{p}={\left\{0,1\right\}}^{p} \end{array}$$

the space of binary strings (responses) of length *p*. For a subject ℓ observed at times *t* = 0, 1, …, *T*, we define

$$D^{\ell }=\left\{D_{0}^{\ell },D_{1}^{\ell },\ldots ,D_{T}^{\ell }\right\},\;D_{t}^{\ell }\in M_{p}$$

the binary multivariate longitudinal data in *p* ≥ 1 variables of subject ℓ. The binary longitudinal data in *p* variables from a population of *n* ≥ 1 subjects observed over time *T* is the set

$$D_{\left[p,n,T\right]}=\{D^{1},D^{2},\ldots ,D^{n}\}.$$

We will only consider subjects with complete data.

Analysis of BMLD *always* involves a fixed variable order where one can use the summary measure of the frequency of response pattern (elements of *M*_*p*_) and perform factor analysis on the longitudinal data [1] or construct Markov models using information of change of time encoded in the matrix of transition probabilities [3]. The method of orbits [9] uses the fundamental information of *frequency of change* of variables and order of variables for analysis. The information of change is used to define a non-autonomous dynamical system from data of each subject, dynamically rearranging order of variables so that most stable least changing variable is eventually placed to the left, but keeping the full information in the original data. Mathematical properties of the map are discussed in [9].

**Fig 1:**
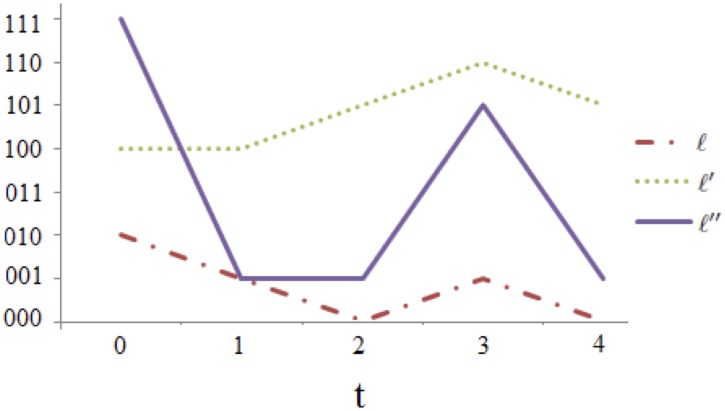
Time series of the three subjects in Table 3.

To explain the orbit method, we illustrate for *p* = 3 variables. Let *Q* = {*Q*_0_, *Q*_1_, *Q*_2_} be a questionnaire and assign index *i* to *Q*_*i*_, *i* = 0, 1, 2. Table 3 illustrates concatenated coded answers to *Q* of three subjects from a sample population. To *Q*_0_, subject ℓ has constant answer 0 while ℓ′ has constant answer 1. On the other hand, ℓ″ has constant answer 1 to *Q*_2_. Observe that this property of subjects having constant answers to certain questions is not trivially illustrated in the time series of the three subjects given in Fig 1.

**Table 3:** Concatenated coded answers of three subjects to questionnaire *Q* = {*Q*_0_, *Q*_1_, *Q*_2_}.

*t*	ℓ	ℓ′	ℓ″
0	010	100	111
1	001	100	001
2	000	101	001
3	001	110	101
4	000	101	001

Suppose we order questions and give more weight to those that least frequently change. As in numbers, we let the digit in the left-most position of the question order be most significant, and digit in the right-most be least significant. Observe that for both ℓ and ℓ′, answer to *Q*_0_ is the most stable (i.e. it is constant), followed by *Q*_1_ (changes once in ℓ and twice in ℓ′), and finally *Q*_2_ as most frequently changing. Then question order for both ℓ and ℓ′ is chosen as *Q*_0_, *Q*_1_, *Q*_2_, which we will denote by 012. Now position lexicographically in increasing order as binary integers the states (responses)

$$\begin{array}{c} 000,001,010,011,100,101,110,111 \end{array}$$ (3)

along an axis, and denote this by *X*_3_. For fixed question order 012, a one-dimensional dynamics on the states in *X*_3_ arises, where answers of subjects ℓ and ℓ′ are visualised jumping from one state to another, particularly staying in the distinct regions 0** and 1**, the left and right half of *X*_3_, respectively. However, different subjects may have different frequencies of change in answer values. Because ℓ″ has constant answer to *Q*_2_, question order for ℓ″ is chosen such that *Q*_2_ is given more weight. In particular, question order for ℓ″ is set to 210.

We recall terms and notations as introduced in [9]. Let

$$\begin{array}{l} p\;:\;\text{number of variables}\\ \ell \;:\;\text{subject from the population}\\ n\;:\;\text{number of subjects}\\ Q\;:\;\text{questionnaire}\\ Q_{i}\;:\;\text{question from}\;Q,\;i\;=\;0,\;1,\;\ldots ,\;p-1\\ f_{i}^{l}\;:\;\text{frequency of change of}Q_{i}\;\text{in data of subject}\;\ell \end{array}$$

**Definition 1** *Given p ≥ 1, the spaces of sequences*

$$\begin{array}{c} X_{p}=\left\{{\left(x_{j}\right)}_{j=0}^{p-1}=x_{0}x_{1}\cdots x_{p-1}:x_{j}\in \left\{0,1\right\}\right\} \end{array}$$

*and*

$$Y_{p}=\{{\left(i_{j}\right)}_{j=0}^{p-1}=i_{0}i_{1}\cdots i_{p-1}:i_{j}\in \{0,1\ldots p-1\},\;i_{j}{}^{\prime }s\;distinct\},$$

*both with the lexicographic ordering of sequences (i.e. as increasing integers) are the fitness axis and significance axis for p variables, respectively. An element x ∈ X_p_ is called a fitness state, and y ∈ Y_p_ a significance state. The space*

$$\begin{array}{c} \begin{array}{ccc} S_{p} & = & \left\{p=\left(x,y\right):x\in X_{p},y\in Y_{p}\right\}\\ & = & X_{p}\times Y_{p} \end{array} \end{array}$$

*is the change space in p variables composed of P = 2^p^ p! states.*

**Fig 2:**
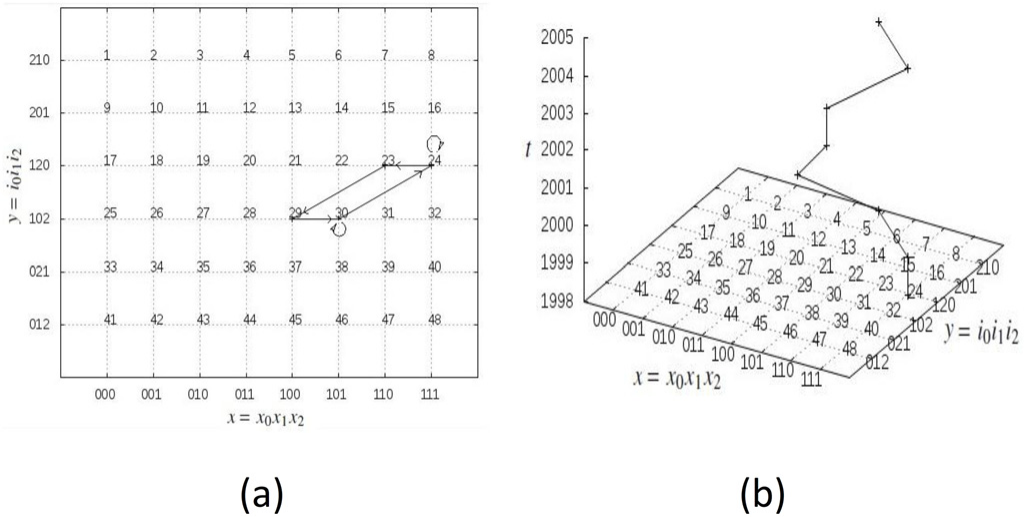
(a) Orbit of subject ℓ staying in subset of *S*_3_ where variable 1 is favourable. (b) Time series of the orbit of ℓ.

For convenience, states in *S*_*p*_ are labeled from 1 to *P* = 2^*p*^ *p*! starting from left to right, top to bottom. The labeled space *S*_*p*_ for *p* = 3 is illustrated in Fig 2. The space *X*_3_ is the sequences in (Eq 3), *Y*_3_ = {012, 021, 102, 120, 201, 210}, and the cardinality of ∣*S*_3_∣ = 2^3^3! = 48.

**Definition 2** *1. Consider subject ℓ. Given a set Q of p ≥ 1 questions, i_j_ ∈ {0, 1, 2, …, p−1}, and Q_i_j__ ∈ Q let*

$$f_{i_{j}}^{\ell }\;=\;number\;of\;times\;Q_{i}{}_{{}_{j}}\;changes\;answer\;in\;data\;of\;\ell \;over\;the\;observation\;period.$$ (4)

*Suppose*

$$\begin{array}{c} f_{i_{0}}^{\ell }<f_{i_{1}}^{\ell }<\cdots <f_{i_{j}}^{\ell }<\cdots <f_{i_{p-1}}^{\ell }. \end{array}$$

*Then the initial question order of ℓ is*

$$\begin{array}{c} y_{0}^{\ell }=i_{0}i_{1}\cdots i_{j}\cdots i_{p-1}. \end{array}$$ (5)

*If $f_{i_{j}}^{\ell }=f_{i_{j+1}}^{\ell },$ use population frequencies $f_{i_{j}}^{n},f_{i_{j+1}}^{n}$ to determine order between *i*_*j*_ and *i*_*j*+1_. If $f_{i_{j}}^{n}=f_{i_{j+1}}^{n}$ and *i*_*j*_ < *i*_*j*+1_, choose question order *i*_*j*_*i*_*j*+1_. Otherwise, choose *i*_*j*+1_*i*_*j*_.*

*2. Given initial question order $y_{0}^{\ell }=i_{0}i_{1}\cdots i_{j}\cdots i_{p-1}$, the initial fitness state of ℓ is*

$$\begin{array}{c} x_{0}^{\ell }=x_{0}x_{1}\cdots x_{j}\cdots x_{p-1} \end{array}$$

*where each *x*_*j*_ is the answer to question *i*_*j*_ in $y_{0}^{\ell }$.*

*3. The initial state of ℓ is $s_{0}^{\ell }=(x_{0}^{\ell },y_{0}^{\ell })\in S_{p}.$*

The algorithm for determining the next states $s_{t}^{\ell }$ (*t* > 0) is as follows:

Step 1: [initial state $s_{0}^{\ell }$] For *t* = 0 and subject ℓ, determine the initial significance state $y_{0}^{\ell }$, followed by the initial fitness state $x_{0}^{\ell }$.
Step 2: [state $s_{1}^{\ell }$] Given initial state $s_{0}^{\ell }=(x_{0}^{\ell },y_{0}^{\ell })$ of ℓ, identify the questions that change answer values at *t* = 1. If there are none, then the next state $s_{1}^{\ell }=s_{0}^{\ell }$. Let
  $$\begin{array}{c} \begin{array}{ccc} x_{j}^{*} & = & \left\{ \begin{array}{cc} 1 & \text{if}\;x_{j}=0\\ 0 & \text{if}\;x_{j}=1. \end{array}\right. \end{array} \end{array}$$
  If both *Q*_*i*_*j*__ and *Q*_*i*_*j*′__ change answers at *t* = 1 and *j* < *j*′, then sequentially swap to the right *i*_*j*_ and *i*_*j*′_ (resp. *x*_*j*_ and *x*_*j*′_) of the question order (resp. answer order), starting with *i*_*j*′_ (resp. *x*_*j*′_). Change *x*_*j*_ to $x_{j}^{*}$ and *x*_*j*′_ to $x_{j\prime }^{*}$, i.e.
  $$\begin{array}{c} \begin{array}{ccc} t=0 & & t=1\\ x_{0}^{\ell }=x_{0}x_{1}\cdots x_{j}\cdot \cdot x_{j^{\prime }}\cdot \cdot x_{n-1} & ⟶ & x_{1}^{\ell }=x_{0}x_{1}\cdot \cdot x_{j-1}x_{j+1}\cdot \cdot x_{j^{\prime }-1}x_{j^{\prime }+1}\cdot \cdot x_{n-1}x_{j^{\prime }}^{*}x_{j}^{*}\\ y_{0}^{\ell }=i_{0}i_{1}\cdots i_{j}\cdots i_{j^{\prime }}\cdots i_{n-1} & ⟶ & y_{1}^{\ell }=i_{0}i_{1}\cdots i_{j-1}i_{j+1}\cdots i_{j^{\prime }-1}i_{j^{\prime }+1}\cdots i_{n-1}i_{j^{\prime }}i_{j} \end{array} \end{array}$$
  This new answer order and question order is the next state $s_{1}^{\ell }=(x_{1}^{\ell },y_{1}^{\ell }).$
Step 3: [edge color] Draw an edge from $s_{0}^{\ell }$ to $s_{1}^{\ell }$. To show direction of transitions between states, color the edge red if transition is from right to left, green if transition is from left to right, and blue otherwise (i.e. same *x*-coordinate).
Step 4: [state $s_{t}^{\ell },t\geq 2]$ Update state $s_{0}^{\ell }$ as $s_{1}^{\ell }$ and time *t* as *t* = 2 in Step 2. Repeat Steps 2 and 3, and iterate until *t* = *T*−1.

**Definition 3** *Let $x_{t}^{\ell }$, $y_{t}^{\ell }$, and $s_{t}^{\ell }=(x_{t}^{\ell },y_{t}^{\ell })$ be the fitness, significance, and state of subject ℓ at time t, respectively. The orbit of ℓ is the sequence of points*

$$\begin{array}{c} 𝓞\left(\ell \right)={\left\{s_{t}^{\ell }\right\}}_{t\geq 0}. \end{array}$$

**Example 1** *Table 4 gives coded data of a subject ℓ to p = 3 questions. Recall that coding of answer is 0 = unfavourable and 1 = favourable according to purpose. The coded answer of ℓ to Q_i_ at time t is denoted by $a_{i,t}^{\ell }$. From (Eq 4), we have $f_{0}^{\ell }=3$, $f_{1}^{\ell }=0,$ and $f_{2}^{\ell }=2$, so initial significance of ℓ is $y_{0}^{\ell }=120$, with corresponding initial fitness $x_{0}^{\ell }=111$. This corresponds to state index 24 in Fig 2(a). No answer changes at t = 1 so $y_{1}^{\ell }=y_{0}^{\ell }$ and $x_{1}^{\ell }=x_{0}^{\ell }$ and state transition from t = 0 to t = 1 is denoted by 24 → 24. Now at t = 2, Q_0_ changes answer so we swap 0 and 1 to the right of $y_{1}^{\ell }$ and $x_{1}^{\ell }$ respectively (note that both are already on the right), and then change answer 1 to 0. Hence, $y_{2}^{\ell }=120$ and $x_{2}^{\ell }=110$, which corresponds to state 23. Columns 3 and 4 give the rest of the fitness and significance states respectively of the orbit. Observe that ℓ has favourable answer to Q_1_ for all times so its orbit 𝓞(ℓ) stays in the subset*

$$\begin{array}{c} \begin{array}{ccc} L(\subset S_{3}) & = & \left\{\left(x,y\right):x_{0}=1\;\mathrm{and}\;i_{0}=1\right\}\\ & = & \left\{21,22,23,24,29,30,31,32\right\} \end{array} \end{array}$$ (6)

*where question i_0_ = 1 is favourable. The longitudinal data of ℓ in S_3_ is visualised as the orbit in Fig 2(a) with its time series illustrated in Fig 2(b).*

**Table 4:** Coded data and orbit of a subject ℓ. The number *a*_*i*, *t*_ is answer to *Q*_*i*_ at time *t*.

*t*	$a_{0,t}^{\ell }$	$a_{1,t}^{\ell }$	$a_{2,t}^{\ell }$	$x_{t}^{\ell }=x_{0}x_{1}x_{2}$	$y_{t}^{\ell }=i_{0}i_{1}i_{2}$	State index
0	1	1	1	111	120	24
1	1	1	1	111	120	24
2	0	1	1	110	120	23
3	0	1	0	100	102	29
4	0	1	1	101	102	30
5	0	1	1	101	102	30
6	1	1	1	111	120	24
7	0	1	1	110	120	23

**Fig 3:**
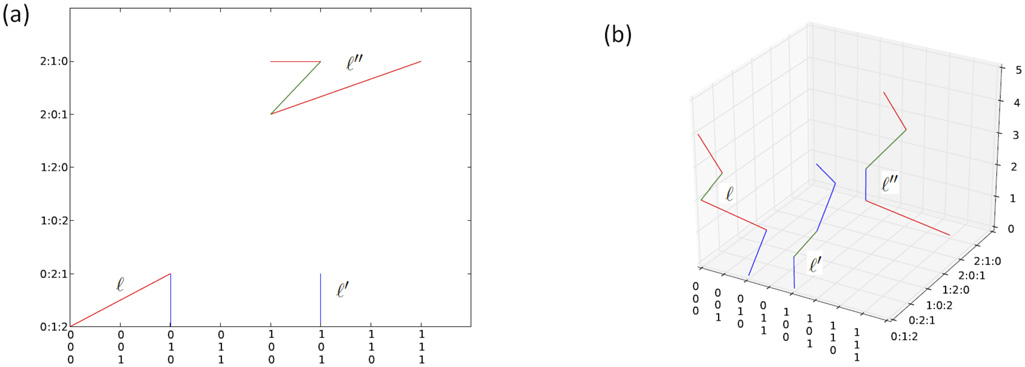
Orbit of the three subjects given in Table 3 (a) in *S*_3_ and (b) over time.

**Example 2** *The orbits of the three subjects in Table 3 in S_3_ and over time are illustrated in Fig 3(a) and 3(b) respectively. Observe that orbit of ℓ stays strictly on the left half of S_3_, and the other two on the right half. Subject ℓ is unfavourable in stable variable 0, while ℓ′ and ℓ″ are favourable in stable variable 0 and variable 2, respectively.*

**Remark 1** *By the 0/1 coding of data, it is reasonable to suppose that the (concatenated) answers composed of unfavourable values 00⋯0 is ‘less fit’ than the answer composed of favourable values 11⋯1. By the weighting of variables, ‘relative fitness’ is made precise so that ordering of elements from the space M_3_ = {0, 1}^3^ of multivariate binary responses has meaning. Because more weight (significance) is given to the left-most position, we can then, for a fixed question order, write x = **0**10 < x′ = **1**00, where most significant variable is unfavourable in x, and favourable in x′. For a fixed question order, we say that 100 is fitter than 010 (or 000, 001). The same argument holds in stating that 110 is less fit that 111.*

Using orbits, the complete *p*-dimensional information of each subject at any moment is coded to a point in the 2-dimensional discrete space *S*_*p*_. No information in data is lost nor approximated as each subject’s orbit has a one-to-one correspondence with the subject’s original data. Clearly, question order of each subject at each time step is monitored. The ordering is selected as frequently changing variables are swapped to the far right (less significant digit of *y*), thus pushing slow changing variables to the left (significant digit of *y*). This ‘swapping-changing-variable-to-the-right’ process exposes clusters associated to stable variables.

**Remark 2** *The time complexity of computation of orbits for n subjects and time T scales like pnT and is feasible for large data. We also note that there are admissible and non-admissible state transitions in S_p_[9], e.g. in Fig 2, a transition that starts at 23 can only end at 23, 24, 29, and 31.*

**Remark 3** *The tendency of a subject to favour a particular state, or subset of states, is clustering in S_p_. The strategy for choosing the initial question order in (Eq 5) places an orbit in its most likely position. This facilitates clustering and is useful for short data sets.*

Note that many households may share an edge (or orbit) in *S*_*p*_. The following definitions are of interest regarding transitions in *S*_*p*_.

**Definition 4** *a. The accumulated number of transitions from state s = (x, y) to s′ = (x′ y′) is called the **density** from s to s′, denoted by d(s, s′).*

*b. The number of orbits at state s at time t is called the **capacity** of state s at time t and is denoted by c_s, t_*.

**Fig 4:**
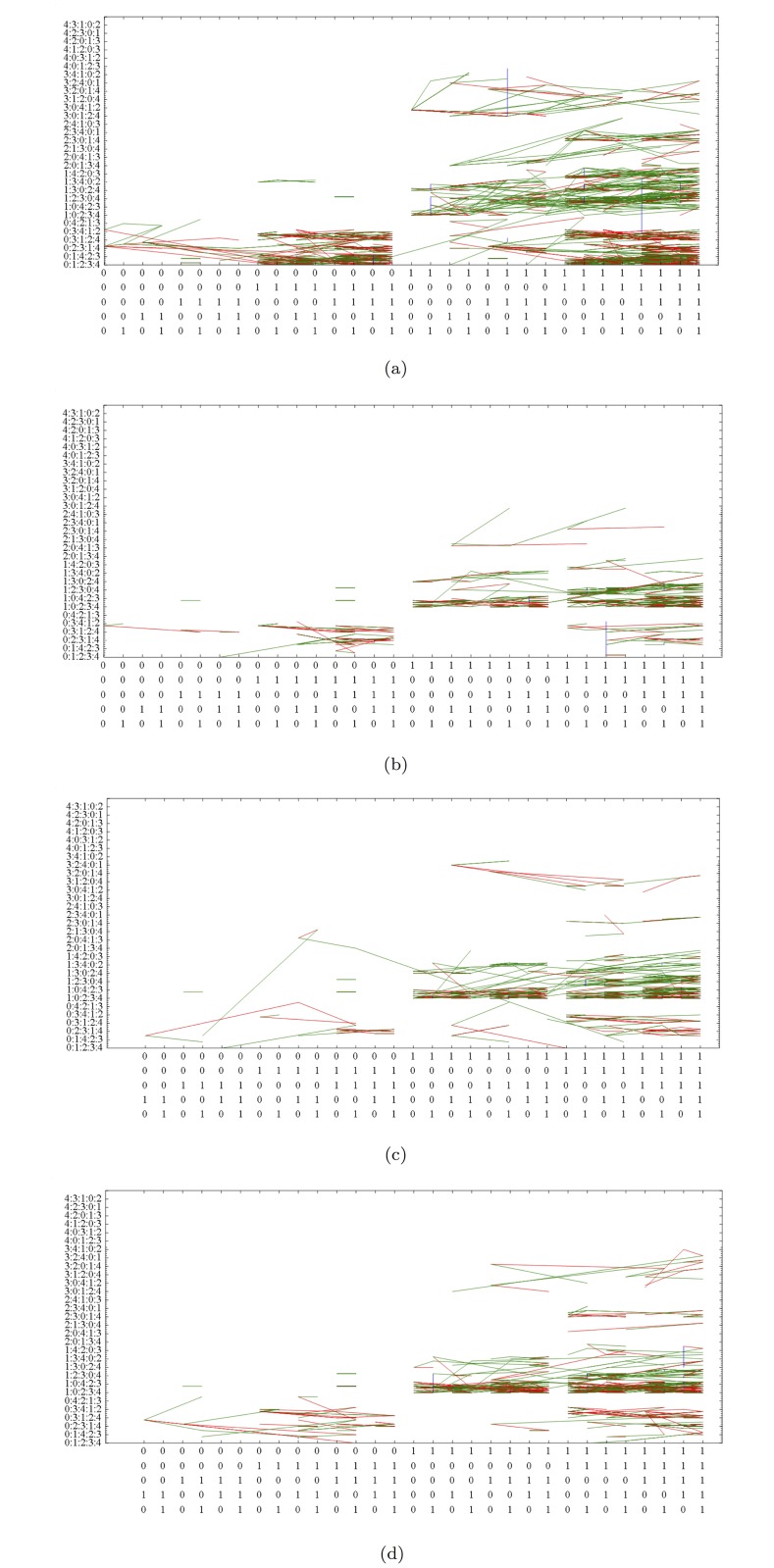
Orbits of (a) SA Rich, (b) SA Poor, (c) MOZ Rich, and (d) MOZ Poor households. Observe clusters formed in regions of each subpopulation. variable 4 (influx) is most frequently changing in all four subpopulations so orbits do not stay in the region where y = 4****. Not all 5! significance states are shown.

**Remark 4** *We can deduce correlation among variables from orbits in S_p_. We explain for p = 3. Using Fig 4, if state transition of orbits most of the time stay in states 1 = (000, 210) and 48 = (111, 012) then we can test for positive correlation among the three variables 0, 1, and 2. If orbits spend most (if not all) of the time in a subset L of S_p_ such that L ≅ S_m_ for some m < p, then there is strong correlation between the first p−m variables constant in S_m_. In Fig 4 for example, if orbits stay strictly in the subset*

$$\begin{array}{c} L=\left\{\left(x,y\right):x=11***,y=01***\right\}≅S_{3}, \end{array}$$

then we can check for (positive) correlation between the first two variables 0 and 1. The asterisk * in x (resp. in y) can take any binary value (resp. any question index except for i).

**Remark 5** *The orbit method is not limited to binary data in p variables. For m-ary valued data, the space S_p_ is composed of same number p! of significance states but now with m^p^ fitness states. For the continuous case, data of each observation are binned, where bins are labelled from 0 to m−1 [18, 19]. For instance, binary coding can be done by assigning 0/1 if variable is above or below a given value, tertiary coding if the variable is in a good/neutral/bad range of values, and so on.*

## Results

### Orbit Results

Household orbits in *S*_5_ for each of the four subpopulations are illustrated in Fig 4. The *x*-axis is composed of 2^5^ = 32 states but not all the 5! = 120 states in the *y*-axis are shown. There are no transitions between the four subpopulations as they are associated to constant variables. Recall that a red edge is used to denote a transition that goes from right to left on the next time step, a green edge for a transition that goes from left to right, and a blue edge a transition that goes to the same fitness state. The percentage of unfavourable answers for each question in each of the four subpopulations is given in Table 5 while the frequencies of answer change are given in Table 6. The frequencies of change for *Q*_0_ (HH) and *Q*_1_ (HS) are low, while *Q*_4_ (IF) is the highest. This means that there is stability in the variables HH and HS in that most subjects will stay in the region where significance is either *y* = 01*i*_2_ *i*_3_ *i*_4_ or *y* = 10*i*_2_ *i*_3_ *i*_4_, *i*_*j*_ ∈ {2, 3, 4}. In addition, there is high activity of the IF variable, which means few transitions where *y* = 4*i*_1_ *i*_2_ *i*_3_ *i*_4_, *i*_*j*_ ∈ {0, 1, 2, 3}. All of this is recognized in Fig 4.

**Table 5:** Percentage of unfavourable = 0 responses in *Q*_*i*_ for each of the four subpopulations.

SA Rich	*Q*_0_ : 17.4%	*Q*_1_ : 11.4%	*Q*_2_ : 20.5%	*Q*_3_ : 21.1%	*Q*_4_ : 22.2%
SA Poor	*Q*_0_ : 33.4%	*Q*_1_ : 10.8%	*Q*_2_ : 13.3%	*Q*_3_ : 44.5%	*Q*_4_ : 23.6%
MOZ Rich	*Q*_0_ : 27.6%	*Q*_1_ : 7.8%	*Q*_2_ : 19.4%	*Q*_3_ : 38.0%	*Q*_4_ : 19.0%
MOZ Poor	*Q*_0_ : 33.5%	*Q*_1_ : 10.3%	*Q*_2_ : 15.0%	*Q*_3_ : 51.4%	*Q*_4_ : 23.5%

**Table 6:** Questions with corresponding frequency of answer change in each of the four subpopulations.

	SA Rich	SA Poor	MOZ Rich	MOZ Poor
*Q*_0_ (HH)	374	78	110	102
*Q*_1_ (HS)	466	53	50	85
*Q*_2_ (HD)	1280	164	216	309
*Q*_3_ (AM)	1334	308	299	405
*Q*_4_ (IF)	3373	580	599	971

**Fig 5:**
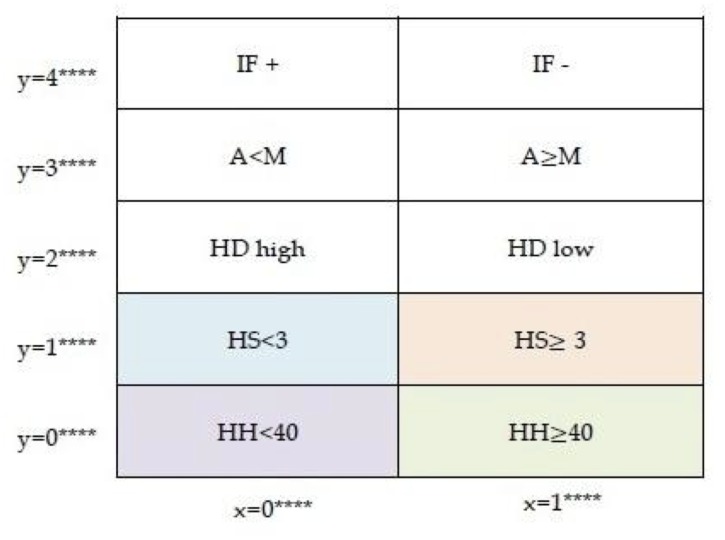
Regions in *S*_5_ determined by the first significant variable. Observed units that often stay in a region determined by one significant variable often experience the property of that region.

There are immediately regions of interest in Fig 4. As an aide in interpreting regions in *S*_5_, we present in Fig 5 the subsets of *S*_5_ determined by the first significant variable *i*_0_. A subject ℓ that spends most of its time in the region where *x* = j****, *y* = i**** means that answer of ℓ to *Q*_*i*_ is least frequently changing, with answer = *j*. The initial state of ℓ is chosen to lie in this region (Definition 5). For example, a subject that stays in the region

$$\begin{array}{c} R_{HH<40}=\left\{p=\left(x,y\right)\in S_{5}:x=0****,y=0****\right\} \end{array}$$

of Fig 5 frequently experiences younger (<40) household head age.

**Fig 6:**
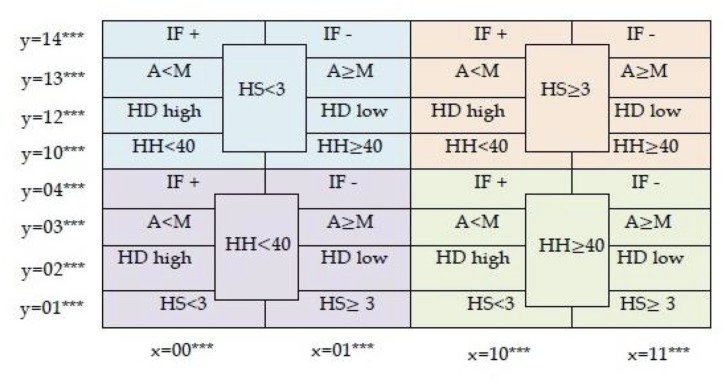
Regions in *S*_5_ determined by the first and second significant variables.

Regions in Fig 5 may be further analysed. A more detailed description of the regions HH≥40, HH<40, HS≥3, and HS<3 is illustrated in Fig 6. In each sub region, the two variables *i*_0_ and *i*_1_ are significant (i.e. less frequently changing answers), with *i*_0_ being more significant. Of course these sub regions may be further subdivided.

In general, we say that a variable *i* is *stable* if orbits cluster in a subset of *S*_*p*_ determined by first significant variable *i*. Regions that are never visited (e.g. those associated to variables IF^+^, IF^−^, and A<M in Fig 4) are termed *holes*. Clusters are contained in regions where the leading significant variable is stable while holes are contained in regions with high activity of the leading variable. By visual inspection of orbits in *S*_*p*_, we can immediately detect stable variables (via clusters) and unstable variables (via holes).

**Fig 7:**
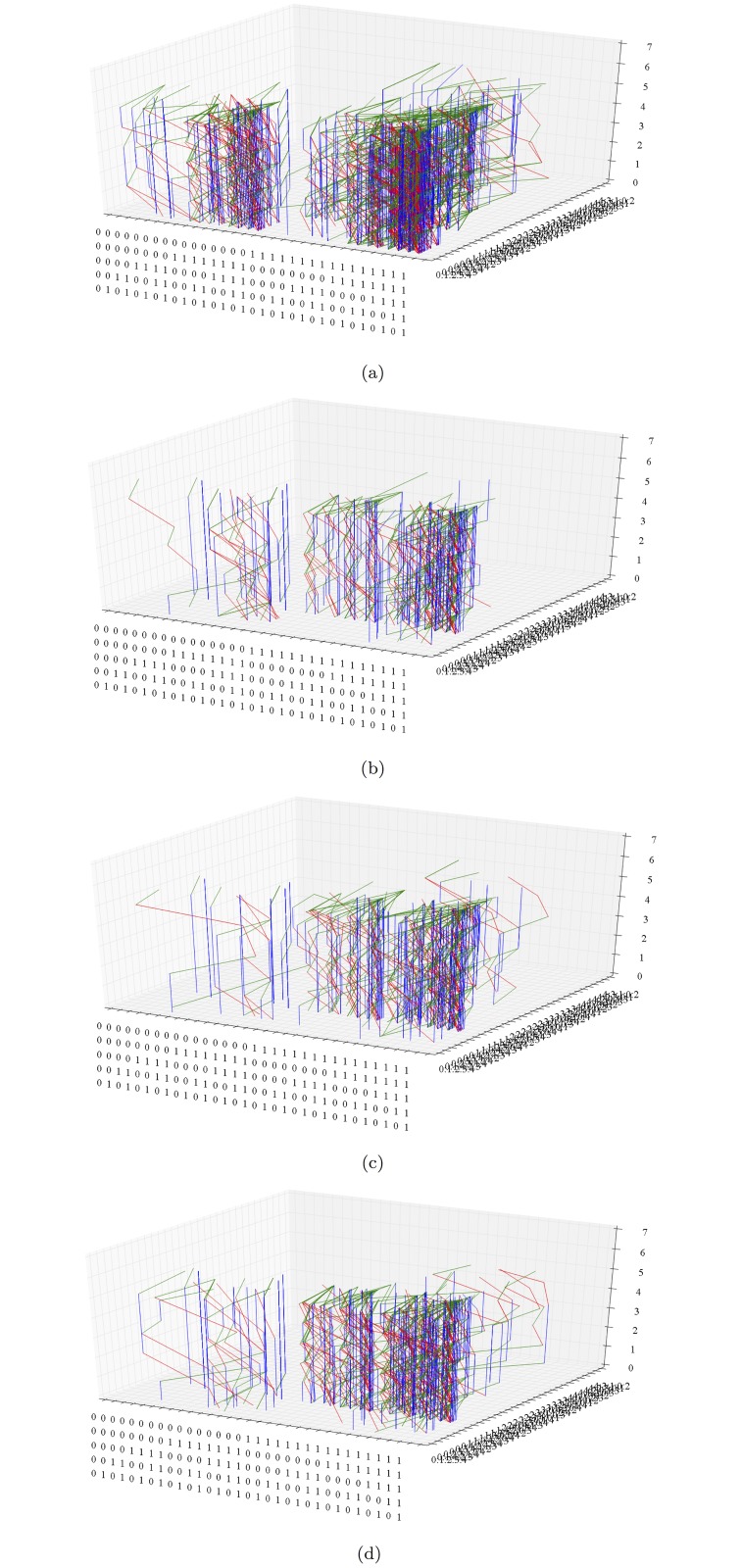
Column structures over clusters of (a) SA Rich, (b) SA Poor, (c) MOZ Rich, and (d) MOZ Poor household orbits in *S*_5_.

Clusters in the right half regions of *S*_*p*_ are fitter than clusters located on the left half of *S*_*p*_ as they are associated to stable leading variable with favourable condition. From the orbits of subpopulations in Fig 4, observe that there are no transitions between the left and right half of *S*_5_ in both SA Poor and MOZ Poor subpopulations. This is verified by Fig 7, the orbits of the four subpopulations, in time. In addition, columnar structures over clusters correspond to variables that are stable over the survey period. Although there are few transitions between clusters, there is considerable activity within each. Household orbits in one cluster may then reasonably be analysed independently of households in other clusters. The time series representation of orbits reveals idle behaviour (sequence of vertical blue edges) that are not always visible in orbits in *S*_5_.

**Fig 8:**
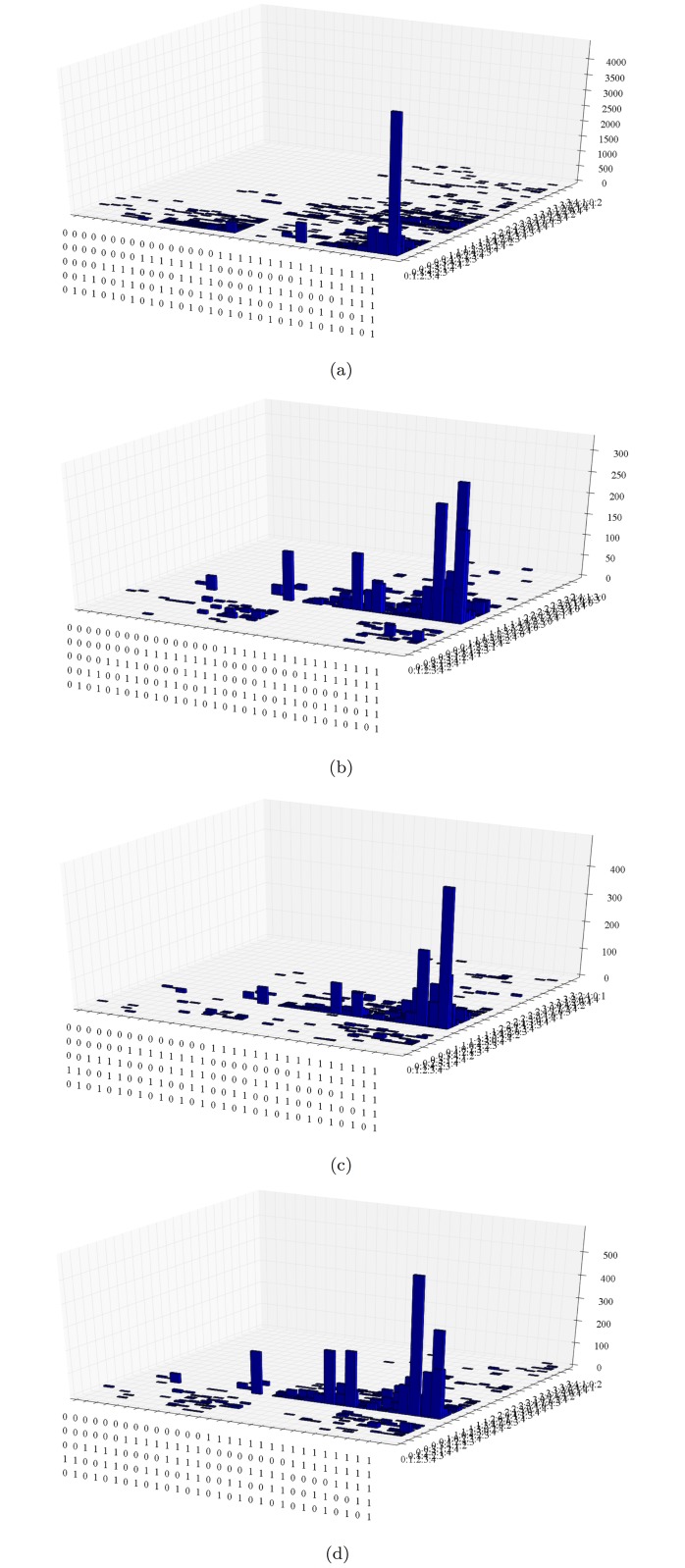
Accumulated number of visits (height of bars) in *S*_5_ of (a) SA Rich, (b) SA Poor, (c) MOZ Rich, and (d) MOZ Poor household orbits.

**Fig 9:**
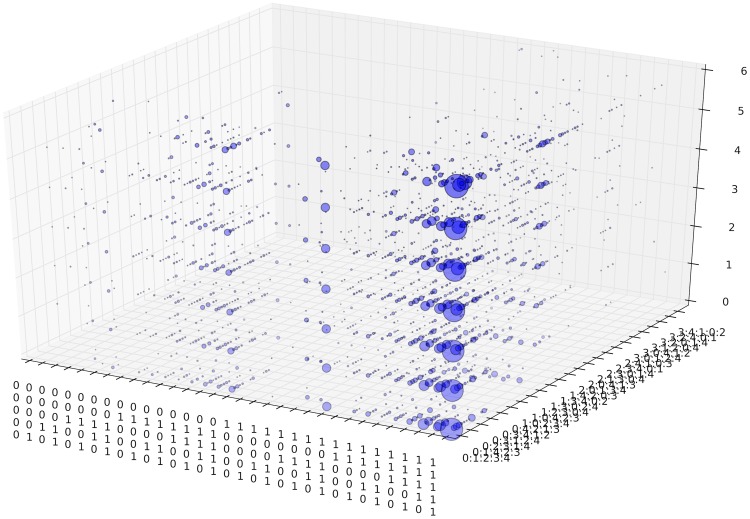
Capacity at each state and each time step in the SA Rich, represented by bubbles.

As observed in Fig 4, some regions in a subpopulation appear denser than those of the other subpopulations (e.g. the region HH<40 appears to be more dense in MOZ poor than in MOZ rich, with the opposite phenomenon for the SA population). We use histograms to denote the accumulated number of visits (i.e. capacity, Defn. 4) to each state in *S*_5_. Fig 8 gives the accumulated capacity in states of *S*_5_, represented by the height of bars. It is immediately noted that there are regions of high and low numbers. Density at each state at each time step can also be computed, and can be represented by bubbles. Fig 9 illustrates this case for the SA Rich subpopulation.

**Fig 10:**
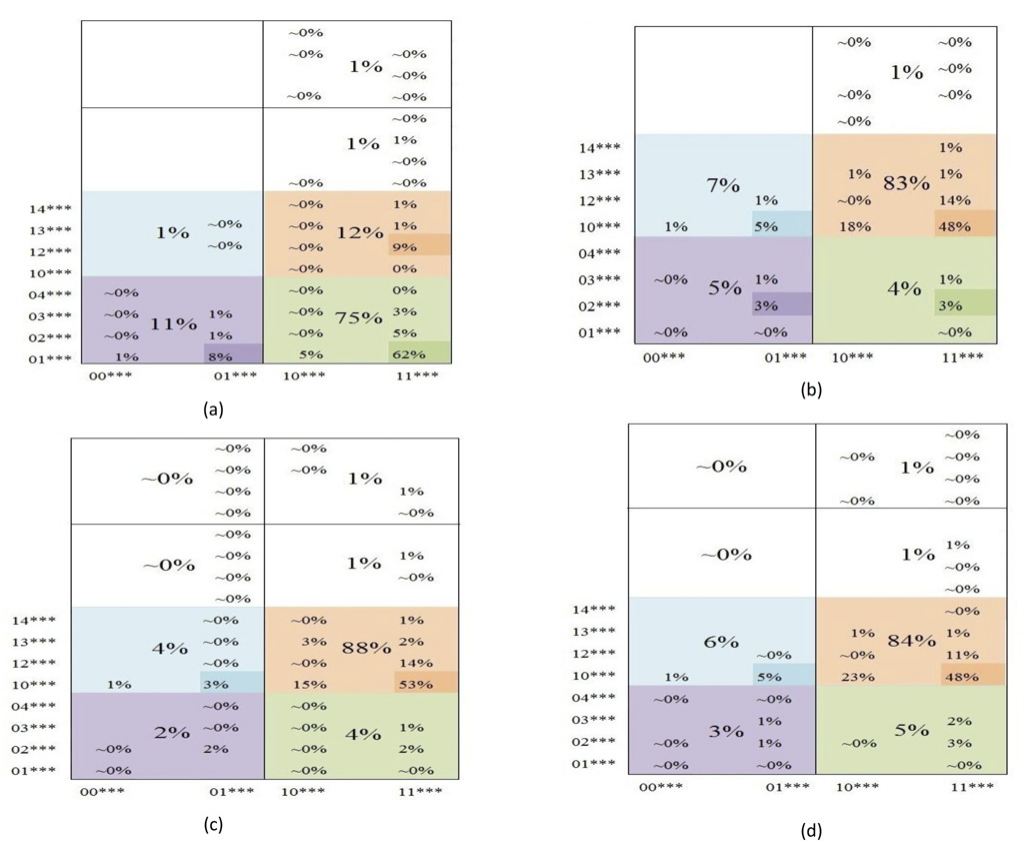
Percentage of visits of (a) SA Rich, (b) SA Poor, (c) MOZ Rich, and (d) MOZ Poor household orbits to regions in *S*_5_ determined by the first and second significant variables.

Fig 10 gives the percentages of visits in regions determined by one and two significant variables. The regions with no percentages are holes. The largest percentage in SA Rich is in the subregion

$$\begin{array}{c} R_{HH\geq 40,HS\geq 3}=\left\{p=\left(x,y\right):x=11***,y=01***\right\} \end{array}$$

associated to older household head and larger household size (62%), with household head more stable. For the other three subpopulations, the largest percentage is in the subregion

$$\begin{array}{c} R_{HS\geq 3,HH\geq 40}=\left\{p=\left(x,y\right):x=11***,y=10***\right\}, \end{array}$$

associated again to older household head and larger household size, but with household size more stable.

**Fig 11:**
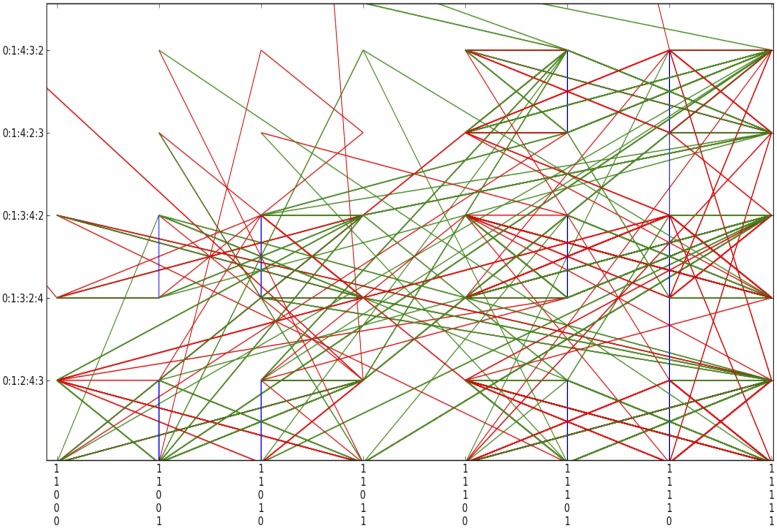
Orbits in SA rich population that cluster in the region *R*_*HH* ≥ 40, *HS* ≥ 3_ where both household head and household size are favourably constant. This is the zoomed region in Fig 4(a) with high percentage of household visit.

**Remark 6** *Population-level information is visible, but detailed individual information may be lost in the cluster. We may zoom into regions of interest (e.g. regions of high percentage of visit) to unclutter the display, as in the SA Rich region R_HH ≥ 40, HS ≥ 3_ illustrated in Fig 11. As for individual orbits, of interest in Fig 4 are those that seem to be ‘outliers’. They can further be analysed e.g. by using interactive techniques such as focusing and brushing, as in dynamic parallel component plots [20].*

**Fig 12:**
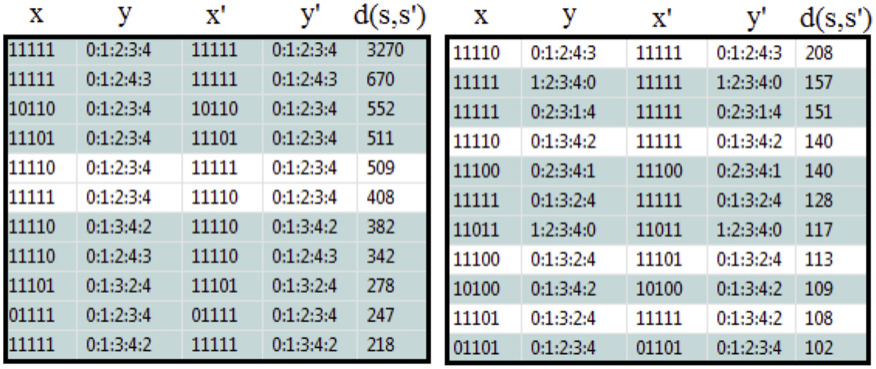
Densities *d*(*s*, *s*′) ≥ 100 from state *s* = (*x*, *y*) to *s*′ = (*x*′, *y*′) in SA Rich households. Highlighted lines are self-transition, i.e. *s* = *s*′.

Regarding Remark 6, we can further analyse orbits from the SA Rich subpopulation. Fig 12 shows dominant accumulated number of transitions ≥ 100 from state *s* = (*x*, *y*) to *s*′ = (*x*′, *y*′) in SA Rich (i.e. density *d*(*s*, *s*′) ≥ 100). Most transitions ‘idle’ at state (11111, 01234) and correspond to household orbits that are constantly favourably in the five variables. As for non-idling transitions, it is dominant between states *s* = (11110, 01234) and *s*′ = (11111, 01234) and involve change in variable 4 (IF) where *s* → *s*′ indicate negative influx, and *s*′ → *s* is non-negative influx.

**Fig 13:**
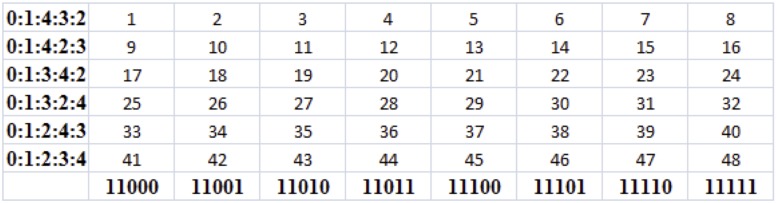
State indices associated to states *s*_*i*_ in the subset *R*_*HH* ≥ 40, *HS* ≥ 3_ of *S*_5_ where both household head and household size are favourably constant.

**Fig 14:**
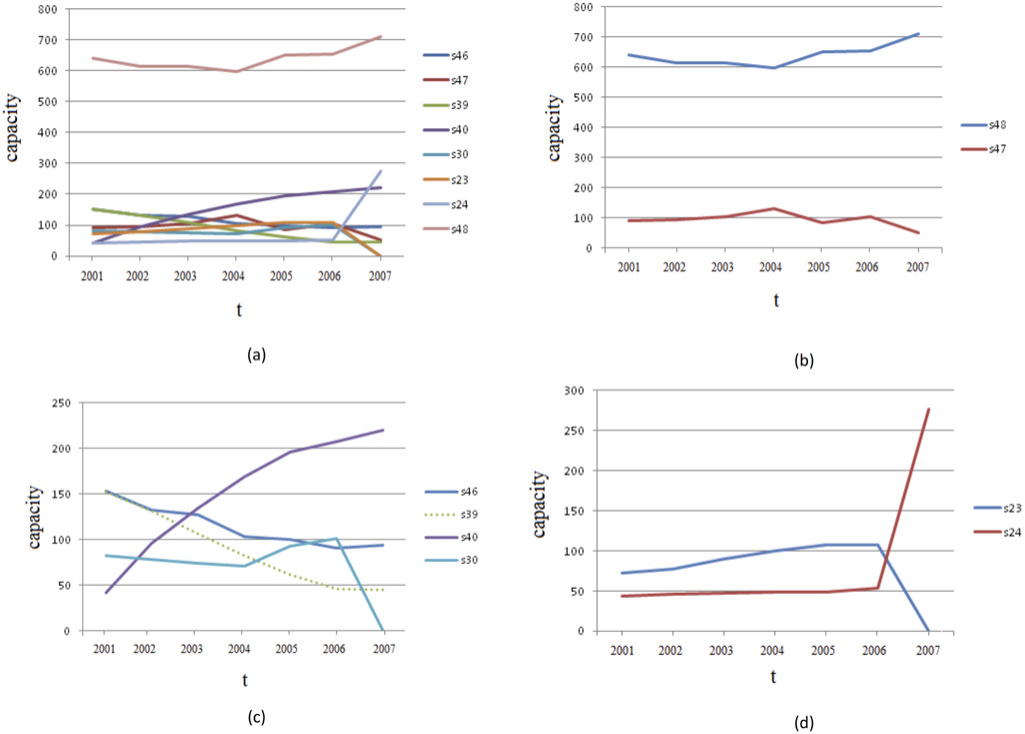
Number of SA Rich household orbits (capacity) at each time step in states (a) 23, 24, 30, 39, 40, 46, 47, and 48, (b) 47 and 48 associated to variable 4 (IF), (c) 39 and 40 associated to variable 3 (AM); 30, and 46, and (d) 23 and 24 associated to variable 2 (HD).

Fig 13 shows the corresponding state indices for the subset *R*_*HH* ≥ 40, *HS* ≥ 3_ of *S*_5_ given in Fig 11. The capacity in SA Rich households at each time step for states with *c*_*s*, *t*_ ≥ 50 is illustrated in Fig 14. We have the following observations:

1. The capacity at state 48 = (11111, 01234) is dominant. This is expected as most orbits idle in this state, as given by the numbers in Fig 12.
2. The capacity graphs for state pairs 48 and 47 = (11110, 01234), and 39 = (11110, 01243) and 40 = (11111, 01243), behave inversely and are almost symmetrical. Note that transition between state pair 47 and 48, and 39 and 40, are associated to change in variable 4 (IF) and 3 (AM) respectively. It is expected that capacity increase in 48 (more individuals migrating into households) result in decrease of capacity in 47. The same argument goes for exchange in capacity of states 39 and 40.
3. Transition between 23 = (11110, 01342) and 24 = (11111, 01342) are associated to change in variable 2 (HD). The capacity graph of 23 (HD = 0) is always above 24, except at *t* = 2007. The sharp increase in 24 (low household death) at this time corresponds to a drop in 23.

### Results Regarding Purpose

We particularly use Fig 8 to draw conclusions with regard to our Purpose as stated in (Eq 1).

1. There is one dominant peak in SA Rich. This occurs at the fully fit state (11111, 01234), where it is most stable in *Q*_0_ = 1 (HH ≥ 40), followed by *Q*_1_ = 1 (HS ≥ 3), and so on. For SA Poor and MOZ Rich/Poor we find fully fit states at (11111, 10234) characterized by stability of HS ≥ 3, followed by HH ≥ 40. We then associate (i) households headed by older adults to larger $\bar{APS}$, and (ii) larger households with lower $\bar{APS}$.
2. The peaks for SA Poor, MOZ Rich, and MOZ Poor are at states
  $$\begin{array}{c} \begin{array}{ccc} i.(11111,10234) & \;\;\;\;\text{iii}.(11111,10243) & \;\;\;\;v.(10111,10234)\\ \text{ii}.(11101,10234) & \;\;\;\;\text{iv}.(11110,10243) & \;\;\;\;\text{vi}.(10101,10234)\\ & & \;\;\;\;\text{vii}.(01110,10234) \end{array} \end{array}$$ (7)
  For spikes at states ii., iv., and vi., unfavourable answer is either in *Q*_0_ or *Q*_3_ (i.e. HH<40 or A<M). We then associate young household heads and less adults to minors to poorer (i.e. not Rich SA) households. Now spike at state vii. which is unfavourable in *Q*_1_ and *Q*_4_ (HS<3 and IF^+^) is also associated to poorer households. The condition of small households should be examined.
3. Spikes at states ii., iv., v., vi., and vii. are identified with relatively stable unfavourable states HH<40, A<M, and HS<3 with IF^+^. We then associate absence of visits to these states with SA Rich, and their presence with the other three subpopulations.
4. For the two dominant peaks at states i. and ii. in MOZ Rich in Fig 8(c), A ≥ M has a higher peak than A<M. This is reversed in MOZ Poor in Fig 8(d). We associate MOZ Poor with a stable, dominant population of households with small adult component.

### Other Methods of Binary Multivariate Longitudinal Data Analysis

We discuss the use of other conventional methods in analysis of BMLD and mention the advantage of using orbits.

#### Markov Chain Model

For *p* binary variables, Markov chain models considers the analysis of change over time measures in *M*_*p*_ = {0, 1}^*p*^. Question order is arbitrarily fixed and a 2^*p*^×2^*p*^ matrix of transition probabilities is constructed [3]. If a fixed question order alone is used for all times and for all subjects (say 012⋯(*p*−1)) in analysing binary multivariate longitudinal data, then some information (e.g. clusters and holes) may not be revealed as orbits overlap in a single row (question order) of *S*_*p*_. For example, the six clusters visible in Fig 4(a) are not resolved in Markov analysis. This phenomenon of ‘unfolding’ states from a general case of a fixed question order is an advantage in analysing orbits in *S*_*p*_. Given the fundamental weighting by frequency of change of variables, it is of great interest that *S*_*p*_ is the space of all possible states to which subjects can change, and also captures change of significant variables. By prioritising slowly changing variables, orbits give a natural spatial ordering of states in *S*_*p*_ by fitness.

#### Generalized Estimating Equation Model

To compare the performance of the conventional statistical model to the deterministic orbit approach we have adopted a generalized estimating equation (GEE) population modelling approach. In [21], the estimation-equation approach is proposed for population average models. It is argued that in general, mixed models involve unverifiable assumptions on the data-generating distribution resulting to potentially misleading estimates and biased inference. We use the quasi-information criterion (QIC) to identify the best working correlation structure to be used for our data [22]. Maximum likelihood based model selection methods, such as the widely used Akaike Information Criterion (AIC), are not directly applicable to the GEE approach [23]. The exchangeable correlation structure proved to be the best when fitted to our data.

Before presenting the GEE model, we note that with regards to the correlated indicators, there is potential co-linearity between the household size and certain other covariates. This is suggested by the marginally high variance inflation factor (VIF) for this variable (*Q*_1_) of ∼ 10 in Table 7. Further, the spearman rank correlation coefficient of 0.68 between *Q*_1_ (household size) and *Q*_4_ (influx) in Table 8 would be cause for further concern. Removing the co-linear effect of *Q*_1_, the GEE model for a binary outcome (APS = 0/1) using a binomial family, logit link function and an exchangeable correlation structure is given in Table 9. The VIF without *Q*_1_ is given in Table 10.

**Table 7:** Variance Inflation Factor.

Variable	VIF	1/VIF
*Q*_1_	10.18	0.0982
*Q*_4_	7.14	0.1340
*Q*_2_	4.73	0.2115
*Q*_0_	3.24	0.3087
*Q*_3_	2.69	0.3714
Mean VIF	5.60	

**Table 8:** Spearman’s Rank Correlation Coefficient.

	*Q*_0_	*Q*_1_	*Q*_2_	*Q*_3_	*Q*_4_
*Q*_0_	1.0000				
*Q*_1_	0.0251	1.0000			
*Q*_2_	-0.0226	-0.0231	1.0000		
*Q*_3_	0.1068	-0.2919	-0.0302	1.0000	
*Q*_4_	0.0381	0.6787	-0.0114	-0.1908	1.0000

**Table 9:** GEE Model removing the co-linear effect of *Q*_1_.

GEE population-averaged model				Number of obs	=	22270
Group variable:		hh_id		Number of groups	=	5567
Link:		logit		Obs per group: min	=	4
Family:		binomial		avg	=	4.0
Correlation:		exchangeable		max	=	5
				Wald chi2(5)	=	483.36
Scale parameter:		1		Prob > chi2	=	0.0000
*Q*_6_ (APS = Rich)	Odds Ratio	Std. Err.	*z*	*P* > ∣*z*∣	[95% Conf. Interval]	
*Q*_0_ (HH ≥ 40)	1.2576	0.0315	9.14	0.000	1.1973	1.3209
*Q*_2_ (HD low)	1.0118	0.0338	0.35	0.724	0.9477	1.0803
*Q*_3_ (A ≥ M)	1.3439	0.0346	11.48	0.000	1.2777	1.4134
*Q*_4_ (IF^−^)	1.2846	0.0375	8.58	0.000	1.2132	1.3603
*Q*_5_ (HN = MOZ)	0.7615	0.0180	-11.51	0.000	0.7270	0.7977
_cons	0.7559	0.0345	-6.13	0.000	0.6912	0.8266

**Table 10:** Variance Inflation Factor (removing *Q*_1_).

Variable	VIF	1/VIF
*Q*_2_	4.43	0.2257
*Q*_0_	3.27	0.3059
*Q*_4_	3.24	0.3089
*Q*_3_	2.66	0.3762
*Q*_5_	1.37	0.7301
Mean VIF	2.99	

**Remark 7** *The GEE model shows that HH ≥ 40, HS ≥ 3, HD low, A ≥ M, IF^−^, and HN = SA are more likely in the rich households. This is consistent with our favourable/unfavourable orbit coding to APS. In addition, the model also informs us that variables associated to holes (not just clusters) in S_p_ should also be analysed. In particular, the Q_4_ (IF) variable (associated to holes) and Q_3_ (AM) variable (associated to very few transitions) appear to be statistically significant and associated to households above the absolute poverty line.*

#### Motion Charts and Heat Maps

**Fig 15:**
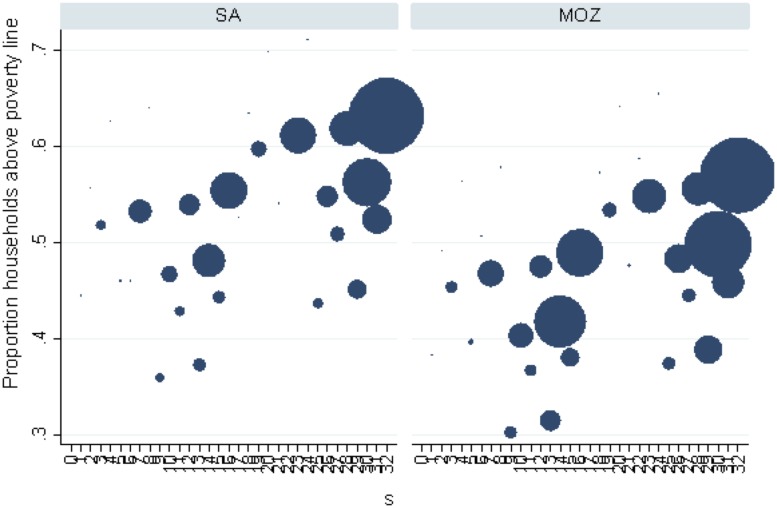
Proportion of households (by nationality and fitness sequence) above poverty line over the survey period 2001–2007.

**Fig 16:**
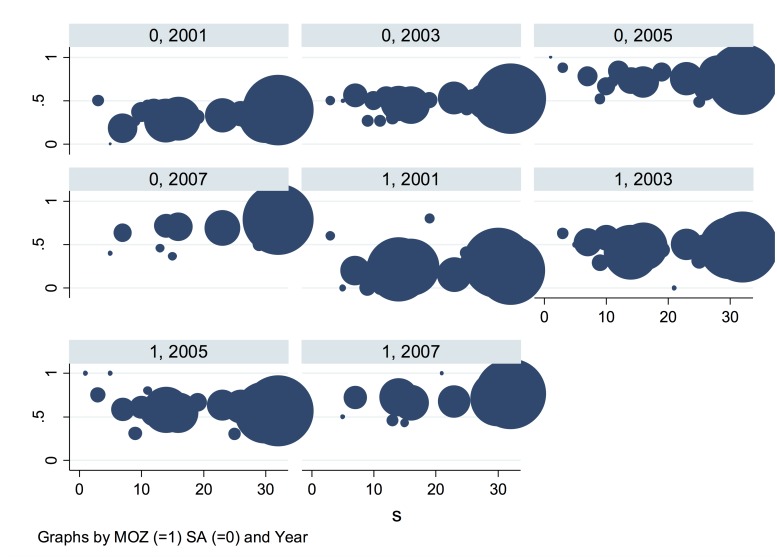
Proportion of households above poverty line by nationality at time (HN, *t*), where HN = 0 = SA, HN = 1 = MOZ, and *t* = 2001, 2003, 2005, 2007.

A motion chart is a dynamic bubble chart that enables the display of large multivariate data with large number of data points [6]. The central object in motion charts is a blob, or in general a 2-dimensional shape, which represents one entity from the dataset. This allows for visualization of the data by using additional dimensions (e.g. time, size and color of the blobs) to show different facets of the data. The dynamic appearance of the data in a motion chart facilitates visual inspection of associations, patterns and trends in multivariate datasets. The problem with motion charts is that for many variables, there is not enough dimensions (e.g. size, shape, color, etc.) to represent different entities. The advantage of using orbits is that adding more variables is easily accommodated by the increase in the number of fitness and significance states in the change space *S*_*p*_. Fig 15 show the proportion of households (by nationality and fitness sequence) above poverty line over the survey period while Fig 16 shows the proportion of households above poverty line by nationality and time, i.e. (HN, *t*), where HN = 0 = SA, HN = 1 = MOZ, and *t* = 2001, 2003, 2005, 2007. The labeling of the fitness states along the *x*-axis is given in Table 11. While this gives a sense of where more households fall with regards to relative poverty probability (stratified by household nationality and then by nationality and poverty line status classification), it does not convey the changing trajectory of households with time.

**Table 11:** Label for answer combination associated to Fig 15 and 16.

s	Answer	s	Answer	s	Answer	s	Answer
1	00000	9	01000	17	10000	25	11000
2	00001	10	01001	18	10001	26	11001
3	00010	11	01010	19	10010	27	11010
4	00011	12	01011	20	10011	28	11011
5	00100	13	01100	21	10100	29	11100
6	00101	14	01101	22	10101	30	11101
7	00110	15	01110	23	10110	31	11110
8	00111	16	01111	24	10111	32	11111

**Fig 17:**
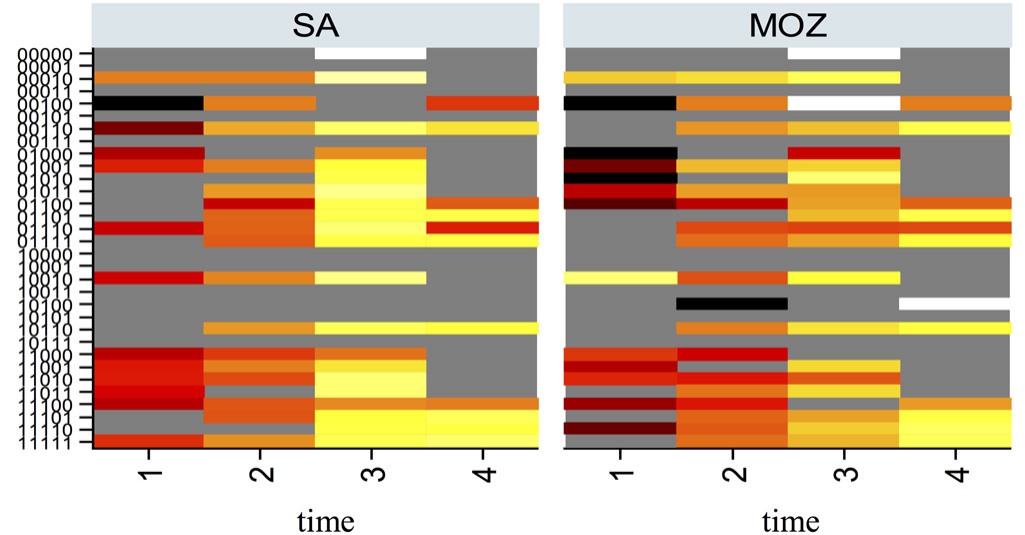
Heat map representing the observed proportion (density) above the poverty line (by nationality) and times 1 = 2001, 2 = 2003, 3 = 2005, 4 = 2007.

The heat map approach illustrated in Fig 17 reflects the observed proportion above the poverty line (by nationality) represented by the amplitude to graph at the point (*x*, *y*), where the fitness sequences are on the *y* axis and the 4 year time points (1 = 2001, 2 = 2003, 3 = 2005, 4 = 2007)are on the *x*-axis. While some differences can be observed by nationality, the clearer visualisation offered by the orbit approach is evident in our opinion. The heat map approach is not without merits (one being easy to implement) and would require more extensive and detailed application to longitudinal data such as ours to fully surmise its utility relative to the deterministic orbit approach.

## Discussion

Using variables pertaining to socio-economic determination, we have illustrated via 2-dimensional orbits the dynamics and patterns of 4 subpopulations in the AHDSS. Stable and unstable variables (in terms of frequency of change) have been identified. The high frequency of change of IF variable (*Q*_4_) in each of the four subpopulations intuitively, is an unfavorable phenomenon because it directly measures instability of household numbers i.e. the rapid flow of individuals into and out of households, within the community. Policies that might stabilize this phenomenon are of interest.

The value of using the method of orbits for analysis of binary multivariate longitudinal data is that it gives a picture of how subjects and the population behave. There are no known methods that show exact visualisation of such data. Orbits can be used as an additional tool for say demographers and social scientist in analysis of data. An additional value of the method is to give insight into possible cause and effect. Presentation of longitudinal data as a time-evolving geometric orbit naturally enables visual identification of possible cause and effect along the orbit (e.g. if only state *i* precedes *j*, then state *i* causes *j*). Using orbits for longitudinal data analysis is fundamentally different from conventional longitudinal statistical models in that it develops visible orbits for fitness states and therefore extracts more information from the data. For instance, the standard statistical model does not give a visual sense of the density of households in a given state, rather just the magnitude of association (odds ratio).

One obvious limitation in using orbits is that it considers only complete data. Extending the method to accommodate missing data is necessary. Tools for (demographic) estimation from limited, deficient and defective data [24] may be used, where longitudinal data does not satisfy the assumption that there is no missing data, or that each variable and each subject is measured at the same times.

The primary confounder we included and stratified on in this analysis was household head nationality. Previous papers [12, 14, 16] on socio-economic status in Agincourt have identified the proximal importance of household nationality as a determinant/confounder for socio-economic status/poverty. Our GEE regression results confirm the importance of this confounder as a determinant of poverty status. As for potential confounders of socio data-economic determination such as occupation and income, they are rarely tracked in the Agincourt HDSS. In addition, given the large amount of missing data for these variables, we would not have be able to apply the orbit theory to the key indicators in the manner presented currently. Within our study period from 2001–2007, the education modules was run only in 2002 and 2006 i.e. not directly captured in the same time points. Mozambicans generally have a significantly lower number of education years compared to South Africans (e.g. [14]) so we believe the nationality would also capture any confounding effects of education status. However we cannot discount any residual confounding influence of occupation, income, and education on our results.

## Supporting Information

S1 Dataset Binary data of the four subpopulations SA Rich, SA Poor, MOZ Rich, and MOZ Poor. (RAR)
